# Supplementary material for: Expression of a Malassezia Codon Optimized mCherry Fluorescent Protein in a Bicistronic Vector
Source: Front Cell Infect Microbiol. 2020 Jul 22;10:367. doi: 10.3389/fcimb.2020.00367 (PMC7387403; doi:10.3389/fcimb.2020.00367)
Supplement: Supplementary file 1 [file Table_1.DOCX]

**Expression of a *Malassezia* codon optimized mCherry fluorescent protein in a bicistronic vector**

**Supplementary material**

| Amino Acid | Codon | Frequency % |
| --- | --- | --- |
| * | TAA | 29.39 |
|  | TAG | 56.46 |
|  | TGA | 14.16 |
| A | GCA | 16.00 |
|  | GCC | 27.63 |
|  | GCG | 40.98 |
|  | GCT | 15.39 |
| C | TGC | 64.41 |
|  | TGT | 35.59 |
| D | GAC | 61.90 |
|  | GAT | 38.10 |
| E | GAA | 28.24 |
|  | GAG | 71.76 |
| F | TTC | 52.82 |
|  | TTT | 47.18 |
| G | GGA | 11.61 |
|  | GGC | 51.64 |
|  | GGG | 13.02 |
|  | GGT | 23.74 |
| H | CAC | 59.96 |
|  | CAT | 40.04 |
| I | ATA | 7.97 |
|  | ATC | 50.54 |
|  | ATT | 41.49 |
| K | AAA | 25.79 |
|  | AAG | 74.21 |
| L | CTA | 7.22 |
|  | CTC | 28.18 |
|  | CTG | 35.85 |
|  | CTT | 13.57 |
|  | TTA | 3.09 |
|  | TTG | 12.10 |
| Amino Acid | Codon | Frequency % |
| M | ATG | 100.00 |
| N | AAC | 59.38 |
|  | AAT | 40.62 |
| P | CCA | 15.36 |
|  | CCC | 26.30 |
|  | CCG | 36.54 |
|  | CCT | 21.79 |
| Q | CAA | 26.10 |
|  | CAG | 73.90 |
| R | AGA | 2.31 |
|  | AGG | 3.38 |
|  | CGA | 8.60 |
|  | CGC | 50.29 |
|  | CGG | 15.97 |
|  | CGT | 19.44 |
| S | AGC | 21.37 |
|  | AGT | 11.16 |
|  | TCA | 9.77 |
|  | TCC | 12.96 |
|  | TCG | 31.64 |
|  | TCT | 13.10 |
| T | ACA | 19.53 |
|  | ACC | 22.05 |
|  | ACG | 44.98 |
|  | ACT | 13.44 |
| V | GTA | 9.63 |
|  | GTC | 27.61 |
|  | GTG | 50.99 |
|  | GTT | 11.76 |
| W | TGG | 100.00 |
| Y | TAC | 64.59 |
|  | TAT | 35.41 |

**Supplementary Table 1**. Relative synonymous codon usage in *Malassezia sympodialis*.


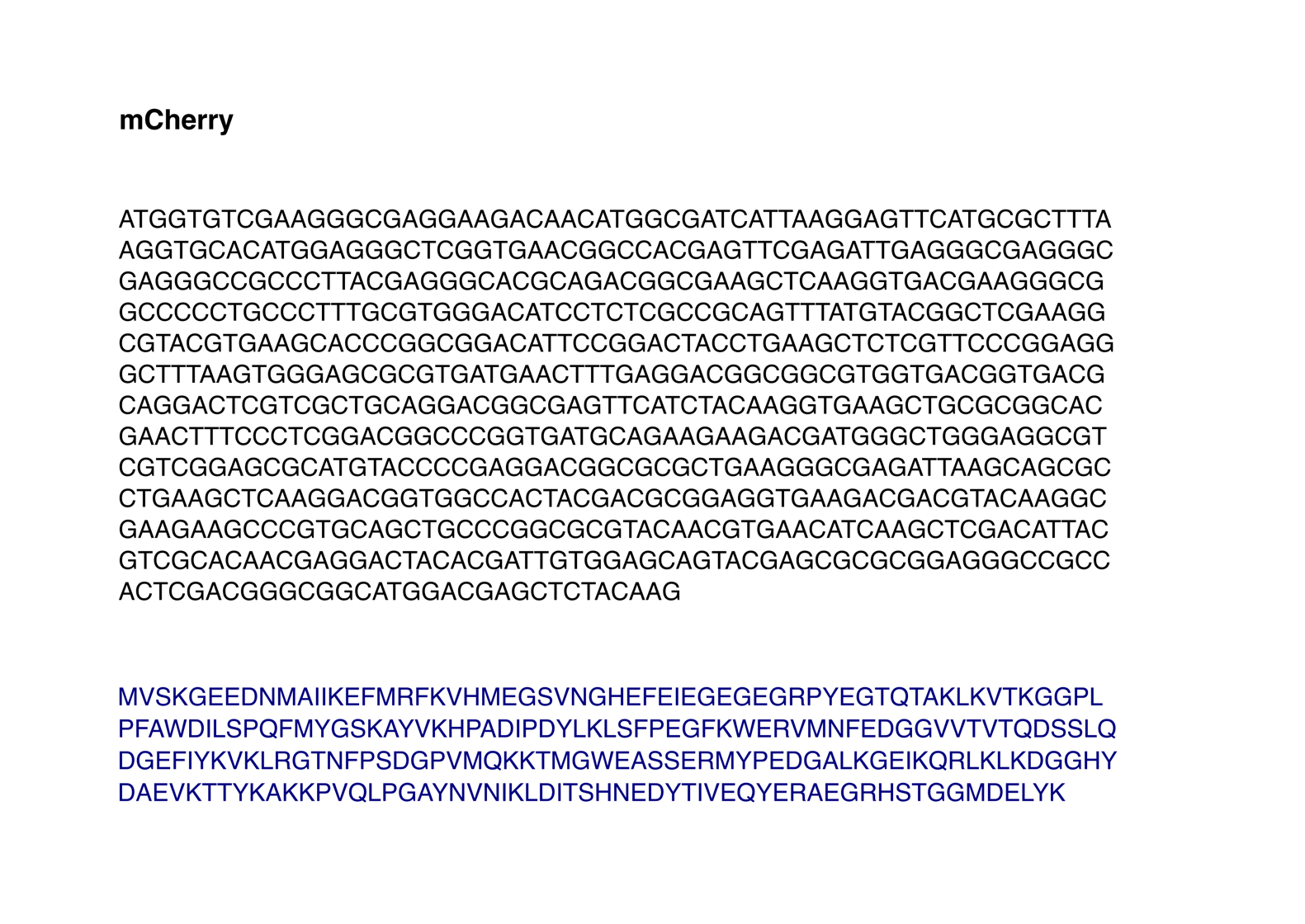


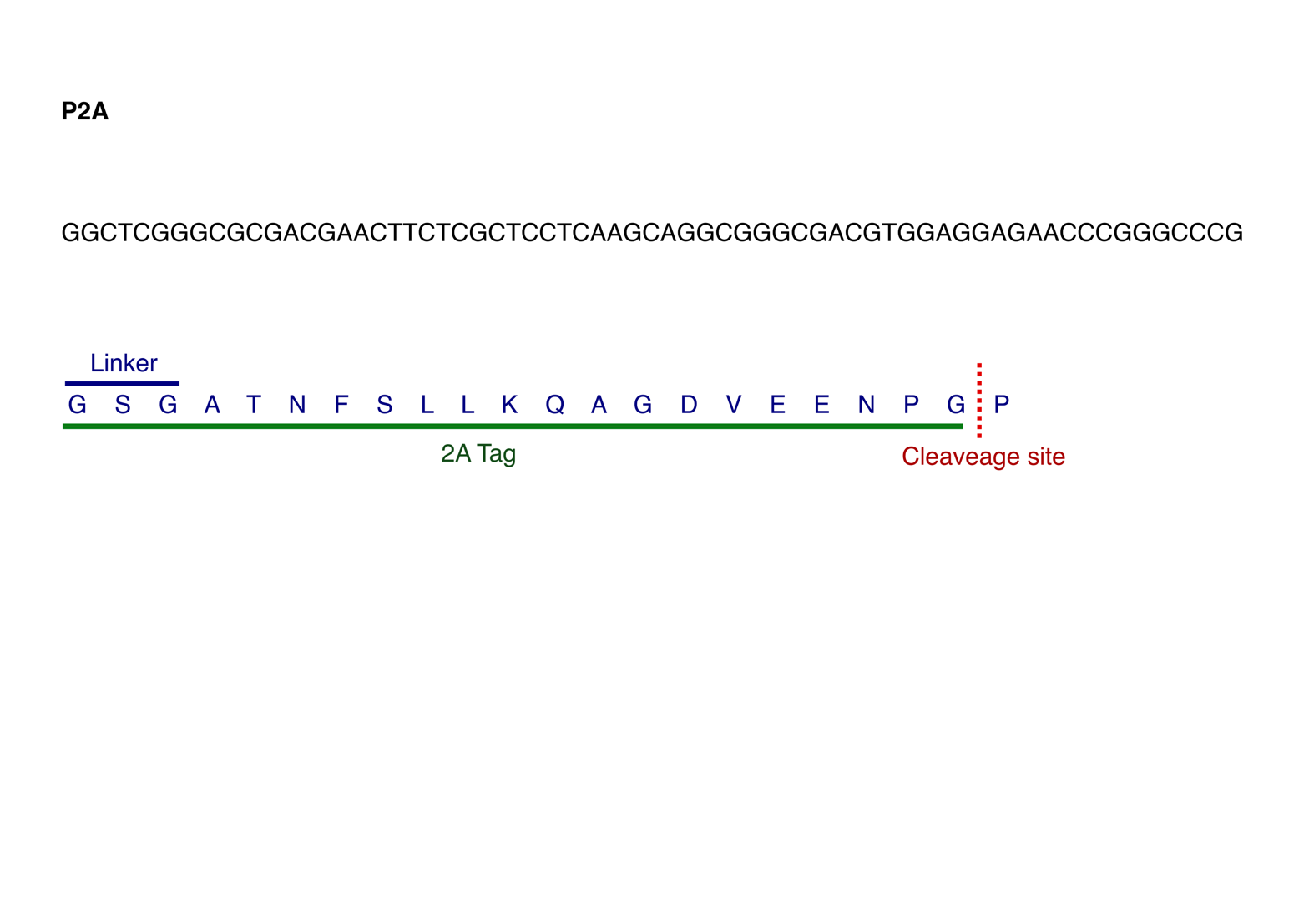


**Supplementary Figure 1.** Codon optimised DNA and protein sequence of mCherry fluorescent protein and porcine teschovirus-1 2A (P2A) pseudo-autolytic peptide.

| *Malassezia furfur* CBS 14141 Chromosome 2, putative CDC25 Phosphatase (143 aa) |
| --- |
| MSFSPPYQYMDADTLASELRRRTQAPNSIAVVDVRDDDYEGGHIVGAIHAPSGTFLTRVDSLVGKELQDYERVVFHCSLSQQRGPKSARIYRETRDAAQAAGRVPTKEQAIFVLRDGFAHFGPKFKNERDLVEDWDEEAWEWR* |

| *Malassezia furfur* CBS 14141 Chromosome 2, hypothetical Adiponectin Protein Receptor (632 aa) |
| --- |
| MGSDVTPSESGSGVSTPPRVSCPSTPNHVHYTPVTAAALAALSDSIEGGPALRASQSSRPPSISDHSEGTHTAAHSAVDAFSLPYWLAYLRSEAARHAHDIDQRMHAILESPDSNDNVLKASISMMTHQLDVVYHALGNLSQRLPMGPSALYDSLPSQSDLSHKLQTLMKDWEQHAHLPHSPLFPQTGPLSFGSLGWNASMEQDATASVPAWSYSSPFGLIPGDWASMVHMPSREQVSDEVHRRLHAFVEQMHSMPARLSQAALPTSLAAWEAPASELLHRVEEKLGEMSEQGRAGASHLVQRANQAVHDVEDVLYQAACELAREGRVLISYQSLPTLWRNNDCIHTGYRFIPVRNWTTLLGSIFQIHNETGNIHTHLSGLILVGALFWFSGSLDSLTTTTDRWIQTLYLLAAAKCLVCSVSWHVMAGCADLNWFQCFACIDYTGISWLVAASLLTLVYNGFYCQPNLIAIYSVGVFLLGTTMGVLPWYPWFDDPKNRTLRISLFVIMALVGLVPFTHGMYLHGFQHMVYFFSPIIPSIASYIAGVVVYALRFPEKYWPGRFDLLGHSHQMWHIAIVLAIALHYRAILLFHKDRFTYSQVDGTCPSFADSLPWSNAAVGGWWPQAGKALGAL* |

**Supplementary Table 2.** Protein sequences of hypothetical genes located both upstream and downstream of the random mutagenesis insertion point in mf::mc-27 strain.
